# Drug‐naïve first‐episode schizophrenia spectrum disorders: Pharmacological treatment practices in inpatient units in Hunan Province, China

**DOI:** 10.1111/eip.13046

**Published:** 2020-09-14

**Authors:** Mengran Zhu, Maria Ferrara, Wenjian Tan, Xingbo Shang, Sumaiyah Syed, Li Zhang, Qilin Qin, Xinran Hu, Robert Rohrbaugh, Vinod H. Srihari, Zhening Liu

**Affiliations:** ^1^ Department of Psychiatry Second Xiangya Hospital of Central South University Changsha China; ^2^ Department of Psychiatry Yale University, School of Medicine New Haven Connecticut USA; ^3^ Program for Specialized Treatment Early in Psychosis (STEP) Connecticut Mental Health Center New Haven Connecticut USA; ^4^ Yale Systems Biology Institute and Department of Biomedical Engineering Yale University New Haven Connecticut USA; ^5^ Department of Neurosurgery Second Xiangya Hospital of Central South University Changsha China

**Keywords:** antipsychotic agents, clozapine, first‐episode schizophrenia, guidelines, polypharmacy

## Abstract

**Aim:**

This study describes antipsychotic prescription patterns for drug‐naïve inpatients diagnosed with first‐episode schizophrenia‐spectrum (FES) disorders and factors associated with practices deviating from China's current guidelines.

**Methods:**

All inpatients aged 7 to 45 years experiencing a first episode of schizophrenia‐spectrum disorder with a duration of untreated illness of less than 18 months and admitted between 1 August 2016 and 1 August 2017 to one of eight psychiatric hospitals in Hunan were included. Demographics, clinical characteristics and prescriptions at discharge were collected from electronic medical records. Logistic regression and random forest methods were used to model relationships between demographic and clinical factors and deviations from China's guidelines.

**Results:**

Of the 602 inpatients included in the study, 598 (99.3%) were prescribed antipsychotics, and no patients were discharged on long‐acting injectable antipsychotics. Polypharmacy (more than one antipsychotic prescribed) was present in 121 (20.2%) participants. Clozapine was prescribed to 45 (7.5%) patients. Adults receiving polypharmacy were more likely to be prescribed high‐dose antipsychotics than those receiving a single antipsychotic. Minors under 13 years of age were more likely to receive polypharmacy and unapproved antipsychotics than those older than 13 years.

**Conclusions:**

Our findings suggest that most of the inpatients were prescribed a single antipsychotic at discharge, consistent with China's guidelines. Minors with FES and patients discharged on polypharmacy and clozapine may require more intense monitoring and management. With the current implementation of China's National Mental Health Working Plan, these results will assist decision‐makers in allocating resources and conducting reforms to facilitate best practice treatment for FES.

## 1 INTRODUCTION

Treatment with antipsychotic medication is a core component of services for first‐episode schizophrenia (FES) (Dixon & Stroup, 2015). Prescribing antipsychotics to this young population is challenging. First, they are experiencing a disorder that may impact their ability to make decisions for their health and well‐being (Parellada et al., 2009). Second, patients with FES are at increased risk of developing side‐effects (Buchanan et al., 2010), which can motivate medication discontinuation (Naber & Kasper, 2000). Therefore, the choice of the first psychopharmacological intervention in drug‐naïve FES patients is a sensitive task, influencing not only adherence to medications but also the course of illness (Perkins et al., 2008).

Currently, FES treatment in Mainland China is provided by psychiatric hospitals, psychiatric departments affiliated with general hospitals and community mental health centres. National Guidelines recommend early and individualized antipsychotic prescription for FES patients, combined with family education, psychotherapy, rehabilitation and vocational training (Chinese Society of Psychiatry affiliated with Chinese Medical Association, 2015).

Most studies conducted in Western countries show trends of increasing and predominant use of second‐generation antipsychotics (SGAs) as first‐line choices for FES (Clavenna et al., 2011; Olfson, King, & Schoenbaum, 2015a, 2015b; Schroder et al., 2017). Few studies have explored treatment practice in China since the 2015 guidelines were released, most have focused on tertiary hospitals, and none have specifically addressed FES.

In this study, we will investigate: (a) the current status of prescription practices for drug‐naïve patients with FES admitted to psychiatric inpatient units in Hunan, China, and (b) demographic and clinical factors associated with deviations from China's published guidelines.

## 2 METHODS

### 2.1 Study setting

This study was carried out in Hunan Province, which has 124 hospitals with psychiatric inpatient units (22 492 beds), serving a population of 67.83 million (Hunan Provincial Bureau of Statistics, 2016). In China, mental health care for schizophrenia is mainly provided in psychiatric hospitals, where medications are the primary intervention, with limited resources for community‐based care. Moreover, such care is unequally distributed across different levels of hospitals. Tertiary hospitals are the best equipped and provide advanced medical investigation and treatment to referrals from primary and secondary hospitals (Zhang et al., 2019).

Eight hospitals (3686 beds in psychiatry units in total; two tertiary and six non‐tertiary hospitals) participated in the study through convenient sampling based on willingness to participate and proximity to the base institution (The Second Xiangya Hospital). It was not possible to collect data from outpatient clinics affiliated with the eight sampling hospitals because medical records were, by default, withheld by the patients.

The data for this study were drawn from a neuroimaging project on neurodevelopment in early schizophrenia (National Natural Science Foundation of China, Grant number: 81561168021), approved by the Ethics Committee of The Second Xiangya Hospital.

### 2.2 Data source and study population

Electronic medical records (EMRs) were retrospectively examined for all patients who met the following criteria: (a) inpatient admission between 1 August 2016 and 1 August 2017; (b) age ≤ 45; (c) discharge diagnosis per the 10th International Classification of Diseases (ICD‐10) (World Health Organization, 1992) of schizophrenia, schizophreniform disorder, acute and transient psychotic disorder, schizoaffective disorder or acute schizophrenia‐like psychotic disorder; (d) duration of untreated illness (DUI) ≤ 18 months; and (e) naïve for exposure to antipsychotics, antidepressants, mood stabilizers and hypnotics‐sedatives. Exclusion criteria were: (a) discharge diagnosis of substance‐induced psychosis or mental retardation; (b) current psychotic disorder due to a general medical condition; (c) history of head trauma; and (d) hospital discharge without a psychopharmacological prescription.

### 2.3 Recommendations in China's Guidelines for Schizophrenia

SGAs except clozapine, are recommended as first‐line treatment in first‐episode schizophrenia (Chinese Society of Psychiatry affiliated with Chinese Medical Association, 2015). Clozapine is recommended for treatment‐resistant schizophrenia, defined as the persistence of symptoms despite ≥2 trials of antipsychotics of adequate dose and duration with documented adherence (Potkin et al., 2020). Antipsychotics recommended for minors (age under 18 years) refer to those approved by the US Food and Drug Administration (FDA) for treating minors with schizophrenia, summarized in Table S1.

### 2.4 Measures and data collection

Data collected from EMRs included gender, age, ethnicity (Han and non‐Han Chinese), residence, diagnosis, tobacco and alcohol consumption, family history of mental illness, insurance (public health insurance only), hospital level (tertiary or non‐tertiary), length of stay (LOS) and psychopharmacological prescription at discharge (generic name and dosage). DUI was computed as the time interval from the first noticeable thoughts or behaviour change related to psychosis to the admission date (Qiu et al., 2019). At discharge, monotherapy was defined as being prescribed one antipsychotic agent; polypharmacy was defined as being prescribed two or more antipsychotics.

For adults (age ≥ 18 years), high‐dose use of antipsychotics was evaluated using prescribed daily dose (PDD) and defined daily dose (DDD). PDD was measured as the dose of antipsychotics prescribed at discharge. DDD is defined as “the assumed average maintenance dose per day for a drug used for its main indication in adults” (WHO Collaborating Centre for Drug Statistics Methodology, 2017). A PDD/DDD ratio greater than 1.5 was defined as high‐dose monotherapy. The sum of PDD/DDD ratio greater than 1.5 across all antipsychotics was defined as high‐dose polypharmacy (Roh, Chang, Yoon, & Kim, 2015).

For minors (age < 18 years), unapproved antipsychotic use was defined as: either the use of antipsychotics unapproved for marketing by the US FDA (eg, amisulpride) or antipsychotics unapproved for specific subpopulations (eg, olanzapine for schizophrenia in 11‐year‐old minors) (detailed in Table S1).

Three deviations from the guidelines were examined: (a) polypharmacy for all subjects, (b) high‐dose antipsychotics in adults and (c) unapproved use of antipsychotics in minors.

### 2.5 Statistical analysis

Means and SDs were calculated for continuous variables. Frequencies and percentages were calculated for categorical variables. Logistic regression was conducted to study linear relationships between factors and likelihood of deviations. A random forest model was built to investigate potential non‐linear relationships between factors and deviations (detailed in supporting information). Age, gender, ethnicity, insurance, hospital levels, LOS, DUI, diagnosis, cigarette and alcohol consumption and family history were included as factors in both models. For model interpretation, odds ratio (OR) and 95% confidence interval (CI) were reported for logistic regressions. Gini‐based variable importance and predicted partial dependence plots (Gareth, Daniela, Trevor, & Robert, 2013; Molnar, 2019) were used to interpret random forests. Statistical significance was set at *P* value < .05. All analyses were processed in R 3.5.3.

## 3 RESULTS

### 3.1 Subjects

There were 602 patients eligible for this study. The majority were male (52.8%), adults (74.3%) and were discharged with a diagnosis of schizophrenia (75.9%) (Table 1).

**TABLE 1:** Demographic and clinical characteristics of the sample

	N	(%)	Mean/median	SD	Range
Age	602	100	25.08/24	9.03	7‐45
Adults (age ≧ 18)	447	74.3			
Male	318	52.8			
Han ethnicity^a^	587	97.5			
Residency in rural areas^b^	390	64.8			
Insurance^c^					
Public health insurance	390	64.8			
No insurance	198	32.9			
Hospitalization in non‐tertiary hospitals	270	44.9			
Length of stay (weeks)	602	100	4.62/3.6	4.33	0.3‐53
Duration of illness (months)	602	100	4.07/2	4.73	0.03‐18
Discharge diagnosis					
Schizophrenia	457	75.9			
Schizophreniform disorder	40	6.6			
Acute and transient psychotic disorders	32	5.3			
Schizoaffective disorder	6	1.0			
Acute schizophrenia‐like psychotic disorder	67	11.1			
Family history of mental illness^d^	75	12.5			
Cigarette consumption^e^	34	5.6			
Alcohol consumption^f^	11	1.8			

^a^ Ethnicity data not available for 1 subject.

^b^ Residency data not available for 26 subjects.

^c^ Insurance data not available for 14 subjects.

^d^ Family history data not available for 6 subjects.

^e^ Cigarette consumption data not available for 13 subjects.

^f^ Alcohol consumption data not available for 13 subjects.

### 3.2 Prescription practice

The most commonly prescribed medications at discharge were oral antipsychotics (99.3%), followed by anticholinergics (33.6%) and mood stabilizers (21.9%) (Table 2). Only 4 patients of 602 (0.7%) were not prescribed antipsychotics at discharge: two were prescribed mood stabilizers, and the other two were prescribed either hypnotics‐sedatives or a combination of hypnotics‐sedatives and antidepressants. No subjects were prescribed long‐acting injectable antipsychotics.

**TABLE 2:** Frequency of prescribed medications at discharge (by class of psychotropic)

	N (n = 602)	(%)
Antipsychotics	598	99.3
Anticholinergics	202	33.6
Mood stabilizers	132	21.9
Beta blockers	91	15.1
Antidepressants	62	10.3
Hypnotics‐sedatives	55	9.1

Within the 598 subjects discharged on antipsychotics, 590 (98.7%) were prescribed at least one SGA, and 24 (4.0%) were prescribed one first‐generation antipsychotic. Most of the patients (79.8%) were on monotherapy, with the two most commonly prescribed antipsychotics being risperidone (40.5%) and olanzapine (40.5%) (Table 3). Among the 121 (20.2%) patients prescribed polypharmacy, the most frequent combination of antipsychotics was that of risperidone and olanzapine (17.4%).

**TABLE 3:** Frequency of antipsychotics prescribed at discharge from the inpatient unit

Generic name	N	(%)
Monotherapy^a^ (n = 477)		
Olanzapine	193	40.5
Risperidone	193	40.5
Aripiprazole	31	6.5
Quetiapine	19	4.0
Amisulpride	13	2.7
Clozapine	12	2.5
Paliperidone	5	1.0
Chlorpromazine	3	0.6
Perphenazine	2	0.4
Sulpiride	2	0.4
Ziprasidone	2	0.4
Iloperidone	1	0.2
Perospirone	1	0.2
Polypharmacy^b^ ^,^ ^c^ (n = 121)		
Olanzapine + Risperidone	21	17.4
Clozapine + Risperidone	14	11.6
Aripiprazole + Olanzapine	13	10.7
Quetiapine + Risperidone	12	9.9
Aripiprazole + Quetiapine	6	5.0
Chlorpromazine + Risperidone	6	5.0

^a^ Monotherapy: being prescribed one antipsychotic agent.

^b^ Polypharmacy: being prescribed two or more antipsychotics.

^c^ For polypharmacy, only the six most frequently prescribed patterns of combined antipsychotics were listed (6 of 28).

Among the 45 (7.5%) patients prescribed clozapine at discharge, 35 of them were adults, and 43 were diagnosed with schizophrenia. Clozapine prescription was more common in non‐tertiary hospitals than tertiary hospitals (χ^2^ = 8.606, *P* = .003) (Table S2) and was more frequently prescribed in antipsychotic polypharmacy (χ^2^ = 81.488, *P* < .001) than monotherapy. Compared to those without clozapine prescription at discharge, patients prescribed clozapine had longer mean LOS (8.02 vs 4.35 mean LOS in weeks, *P* < 0.001).

### 3.3 Deviations from China's guidelines

#### 3.3.1 Deviations from China's guidelines for polypharmacy prescription

**FIGURE 1:**
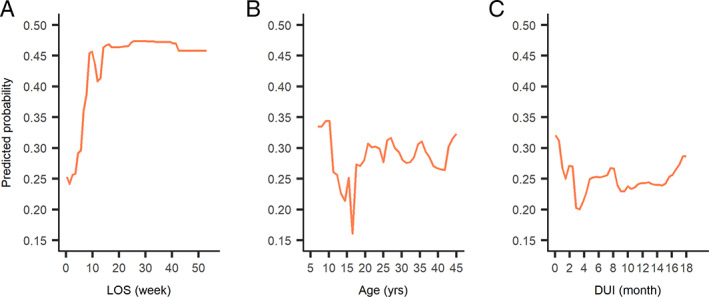
Partial dependence plot of predicted probability of receiving polypharmacy based on length of stay (LOS) (1A), age (1B) and duration of untreated illness (DUI) (1C)

Polypharmacy was prescribed for 99 of 447 (22.1%) adults and 22 of 155 (14.2%) minors (Table S3). As shown in Table 4, adults (OR = 1.95, 95% CI 1.14‐3.34) and patients with longer LOS (OR = 1.09, 95% CI 1.03‐1.14) were significantly more likely to receive polypharmacy. In random forest models (Figure S1), LOS, age and DUI were the three most important factors predicting polypharmacy prescription. As shown in Figure 1, the likelihood of being prescribed polypharmacy increased as LOS increased within 2 months of LOS but did not increase further with LOS greater than 2 months. Patients younger than 11 years old had higher predicted probability of receiving polypharmacy than any other age group. No clear trends could be drawn about the effect of DUI on the predicted probability of polypharmacy prescriptions.

**TABLE 4:** Multinomial logistic regression of different types of deviations from guidelines

Type of deviation	OR	95% CI		*P*
Polypharmacy prescriptions (n = 568^a^)				
Adult	1.95	1.14	3.34	.015*
Length of stay	1.09	1.03	1.14	.001**
Diagnosis of acute and transient psychotic disorders	0.19	0.03	1.01	.051
High‐dose prescriptions (n = 420^b^)				
Age	0.96	0.93	0.99	.008**
Non‐tertiary hospitalization	0.38	0.24	0.59	<.001***
Polypharmacy	3.56	2.17	5.86	<0.001***
Unapproved prescriptions in minors (n = 149^c^)				
Age	0.46	0.34	0.63	<.001***
Length of stay	1.29	1.09	1.52	.003**
Polypharmacy	6.22	1.92	20.31	.002**

Abbreviation: CI, confidence interval; OR, odds ratio.

^a^ Not included 30 observations with missing values.

^b^ Not included 22 observations with missing values.

^c^ Not included 6 observations with missing values.

* *P* < .05.

** *P* < .01.

*** *P* < .001.

#### 3.3.2 Deviations from China's guidelines for dosage

**FIGURE 2:**
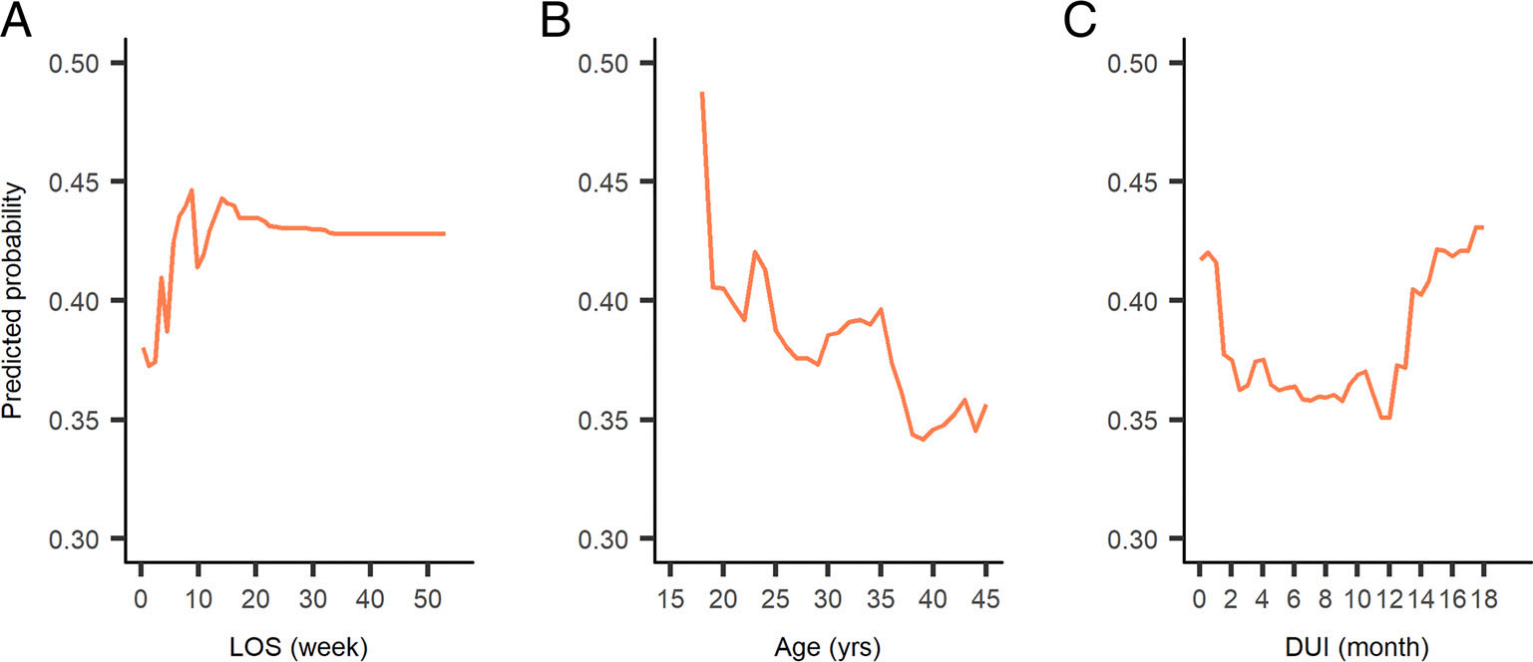
Partial dependence plot of predicted probability of receiving high‐dose antipsychotics based on length of stay (LOS) (2A), age (2B) and duration of untreated illness (DUI) (2C) in adults

Of 443 adults, 147 (33.2%) were prescribed high‐dose antipsychotics (Table S3). As reported in Table 4, subjects with non‐tertiary hospitalization (OR = 0.38, 95% CI 0.24‐0.59, compared to tertiary hospitalization) were less likely to be prescribed high‐dose antipsychotics. Patients of younger age (OR = 0.96, 95% CI 0.93‐0.99) and receiving polypharmacy (OR = 3.56, 95% CI 2.17‐5.86, compared to monotherapy) were more likely to receive high‐dose antipsychotics. Olanzapine, quetiapine and amisulpride were dosed higher when used as mono‐ vs polypharmacy (Table S4). In random forest models (Figure S2), LOS, age and DUI were the three most important factors predicting high‐dose use. As shown in Figure 2, the likelihood of receiving high‐dose antipsychotics increased as LOS increased within 9 weeks of LOS and but did not increase further after 9 weeks of LOS. Younger patients were more likely to receive high‐dose antipsychotics. Patients with shorter DUI (<2 months) and longer DUI (>12 months) were more likely to receive high‐dose antipsychotics.

#### 3.3.3 Deviations from China's guidelines for unapproved use of antipsychotics

**FIGURE 3:**
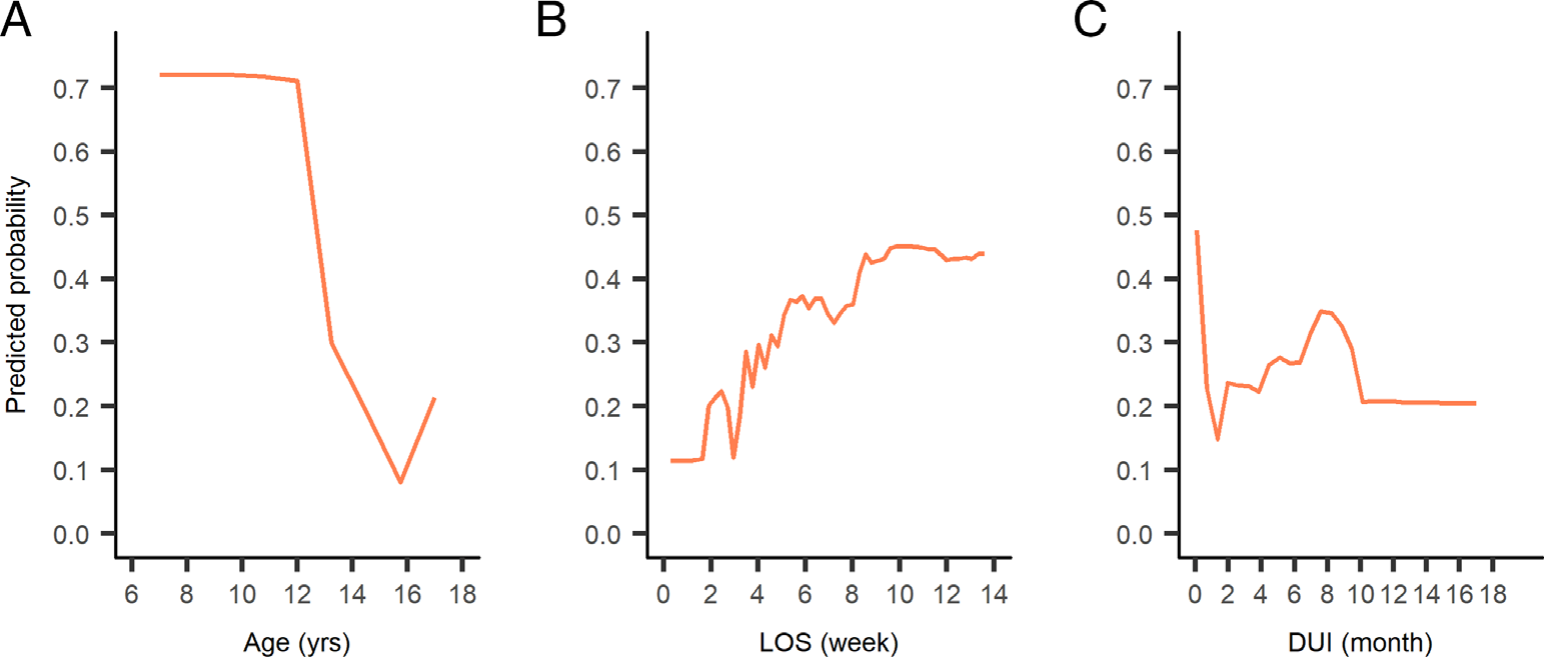
Partial dependence plot of predicted probability of receiving unapproved antipsychotics based on length of stay (LOS) (3A), age (3B) and duration of untreated illness (DUI) (3C) in minors

Among 155 minors, 36 (23.2%) received antipsychotics with unapproved use (Table S3): 25% of these were younger than 12 years old. As shown in Table 4, minors with younger age (OR = 0.46, 95% CI 0.34‐0.63), longer LOS (OR = 1.29, 95% CI 1.09‐1.52) and polypharmacy (OR = 6.22, 95% CI 1.90‐20.31 compared to monotherapy) were more likely to demonstrate unapproved use of antipsychotics. In a random forest model (Figure S3), age, LOS and DUI were the three most important factors predicting unapproved use. As shown in Figure 3, the predicted probability of unapproved use was higher for minors under 13 years of age than any other ages, minors with longer LOS and those with shorter DUI (<2 months).

## 4 DISCUSSION

This is the first study to explore treatment practices for inpatients with drug‐naïve FES disorders in China after the release of the latest national guidelines.

Most patients, at discharge, were prescribed a single antipsychotic, which is consistent with guideline recommendations. None of the patients were prescribed long‐acting injectable antipsychotics. One in five patients was prescribed polypharmacy. Among adults, one‐fourth were prescribed high‐dose antipsychotics. In minors, one‐fifth received an unapproved antipsychotic at discharge. Patients with longer LOS had a higher likelihood of being prescribed polypharmacy. Within adults, high‐dose antipsychotics were more likely to be prescribed to younger adults and to those receiving polypharmacy (compared to monotherapy). Unapproved antipsychotic prescription in minors was more likely to be directed at those below 13 years of age and in those with longer LOS.

### 4.1 Prescription practice

As expected, the majority of patients were discharged with a prescription for antipsychotics, and most received SGAs, mainly risperidone and olanzapine, which is consistent with practice in the United States (Robinson et al., 2015). Although there is a lack of robust evidence supporting the preferential use of olanzapine or risperidone vs other SGAs (Komossa et al., 2010; Temmingh, Williams, Siegfried, & Stein, 2018), this may be explained by greater medication availability and prescriber familiarity as both drugs have been available in China since the late 1990s. However, prescribing olanzapine to first‐episode patients as first‐line treatment has become controversial (Dixon & Stroup, 2015). Increasing evidence shows significant severe adverse metabolic effects caused by olanzapine, which can increase risks for cardiovascular diseases and diabetes (Correll et al., 2014; Dixon & Stroup, 2015; Tek et al., 2016). Close monitoring and management of adverse metabolic effects are needed after discharge to reduce long‐term risks and to maintain medication adherence (Curtis, Newall, & Samaras, 2012; Ferrara et al., 2015). This would require an increased engagement of community‐based mental health care services, currently not available in China.

None of the patients were prescribed long‐acting injectable (LAI) antipsychotics at discharge. This may be related to China's guidelines that recommend LAI for patients only with poor medication compliance or multiple relapses, in contrast to evidence showing benefits earlier in the course of treatment (Stahl, 2014). Alternatively, higher costs, reduced availability in non‐tertiary hospitals or psychiatrists' ambivalent or negative attitudes toward LAIs may influence patient acceptance of these medications (Liu et al., 2015; Weiden et al., 2015).

In our study, 7.5% of FES patients were prescribed clozapine. As the prevalence of treatment‐resistant schizophrenia is estimated to be 20% among FES (Demjaha et al., 2017), this suggests clozapine underutilization, consistent with previous studies (Tang et al., 2016; Thien et al., 2018). Additional training on the safe and optimal use of clozapine may be helpful.

### 4.2 Deviations from China's guidelines

In our sample, the prevalence of polypharmacy (20.2%) is higher than that in the United States (11.0%) (Robinson et al., 2015) and United Kingdom (7.4%) (Tungaraza et al., 2017). China's guidelines discourage polypharmacy in keeping with the lack of robust evidence of effectiveness over monotherapy (Fleischhacker & Uchida, 2014). In addition, prescribing more than one antipsychotic may increase adverse effects and medical costs (Putignano, Clavenna, Reale, & Bonati, 2019). Auditing the most difficult cases in routine workflows may guide quality improvement approaches to promote adherence to current guidelines (Severi et al., 2018).

Both random forest and logistic regression models showed that longer LOS was associated with the higher likelihood of polypharmacy prescription, which is consistent with a previous study (Saldaña et al., 2014). As LOS has been shown to be influenced by multiple factors, including patients' disease severity, prescriptions during hospitalization and insurance type (Baeza, da Rocha, & Fleck, 2018), more information is needed to explain the relationship between LOS and polypharmacy at discharge.

Among adults, one‐third were discharged with high‐dose antipsychotics, a known risk factor for side‐effects (Centorrino et al., 2004), without evidence for improved efficacy (Mace & Taylor, 2015). Lower doses of individual antipsychotics were used within polypharmacy compared to monotherapy; however, patients receiving polypharmacy were more likely to be prescribed high‐dose antipsychotics vs those receiving monotherapy. This can place a greater burden of monitoring and management of side‐effects upon already under‐resourced outpatient services. Compared to hospitalizations in non‐tertiary facilities, patients admitted in tertiary hospitals were more likely to receive high‐dose antipsychotics. This finding might reflect the admission or transfer of patients with more severe illnesses to tertiary hospitals.

In minors, the prevalence of unapproved use of antipsychotics (23.2%) is below the lower limit of the range reported in previous studies (36.0%‐93.2%; Putignano et al., 2019) but is still clinically significant. Minors below 13 years of age had higher odds of receiving unapproved prescriptions. This prescription practice could be a consequence of limited studies informing evidence‐based treatment of childhood schizophrenia and limited antipsychotics approved for minors under 13 years of age (Christian et al., 2012). Our finding—that 25% of 36 minors who were prescribed unapproved antipsychotics were younger than 12 years old—is even more significant if we consider that only chlorpromazine is recommended by FDA for children under 12 years old (Christian et al., 2012). When there is a scarcity of evidence‐based recommendations, unapproved use can depend on the attitudes and competence of psychiatrists (Putignano et al., 2019). Moreover, unapproved medications are more likely to be implicated in adverse reactions (Brauner, Johansen, Roesbjerg, & Pagsberg, 2016), and minors have more variable antipsychotic pharmacokinetics (Caccia, 2013). These factors make unapproved prescriptions more problematic. Further research is needed to better guide prescription practices in the youngest patients.

### 4.3 Strengths and limitations

As the vast majority of mental health care in Hunan is provided by inpatient facilities, our study provides a good representation of current practice and highlights specific gaps in guideline implementation. Education, training and research, as well as improved monitoring and management systems after discharge, will be needed to deliver guideline concordant care.

This study combined, for the first time, logistic regression and random forest to model the relationship between factors and deviations from China's guidelines. The two models gave similar prediction trends. In addition to the linear relationships modelled by logistic regression, our further analysis using random forest revealed that LOS had a significant impact on all three deviations from China's guidelines.

This study had several limitations. The convenience sample may have limited representativeness. However, the sample included the two best‐rated tertiary hospitals in Hunan, which suggests that greater deviations from the guidelines might exist in the out‐of‐sample hospitals. Data on symptom severity at admission were not collected, which makes it difficult to investigate the rationale for prescription practice and deviations from guidelines. Moreover, data on clinical outcomes at discharge were not collected, which limits the interpretation of the results. In addition, as the sample was drawn from inpatient psychiatric hospitals, with more acute, aggressive and rapidly escalating clinical presentations, the findings may not generalize to outpatient facilities. Further investigation of FES care in outpatient services is necessary.

## 5 CONCLUSIONS

Our study showed that, at discharge, most of the inpatients diagnosed with first‐episode schizophrenia were prescribed antipsychotic monotherapy, in compliance with current guidelines. Our findings suggested potential challenges to optimal prescription practices, especially for minors and treatment‐resistant schizophrenia. More research, education and training are warranted to verify and resolve these challenges. Given the ongoing implementation of China's National Mental Health Working Plan, these results provide a useful representation of current inpatient practice in China and can inform resource allocation towards optimal treatment of first‐episode psychosis.

## CONFLICT OF INTEREST

Vinod Srihari has served on an Advisory Board for Takeda, Inc.

## Supporting information

**Data S1.** Supporting information.

**Figure S1.** Variable importance plot in predicting polypharmacy prescription.

**Figure S2.** Variable importance plot in predicting high close prescription in adults.

**Figure S3.** Variable importance plot in predicting unapproved use of antipsychotics in minors.

**Table S1.** Approved age range of antipsychotics for schizophrenia in China's guidelines.

**Table S2.** Differences of factors in clozapine prescription.

**Table S3.** Frequency of prescriptions deviating from guidelines (Chinese Society of Psychiatry affiliated with Chinese Medical Association, 2015).

**Table S4** Dosage of single antipsychotic in monotherapy^a^ and polypharmacy^b^.

## Data Availability

The data that support the findings of this study are available on request from the corresponding author. The data are not publicly available due to privacy or ethical restrictions.
