# Supplementary material for: Drug‐naïve first‐episode schizophrenia spectrum disorders: Pharmacological treatment practices in inpatient units in Hunan Province, China
Source: Early Interv Psychiatry. 2020 Sep 14;15(4):1010–8. doi: 10.1111/eip.13046 (PMC8359180; doi:10.1111/eip.13046)
Supplement: Supplementary file 1 — Data S1. Supporting information. [file EIP-15-1010-s008.docx]

**Details in model building**

The complete dataset was randomly divided into a training set and a test set (7:3). All models were fit to the training set, and the performance of a specific model was evaluated by its error rate when applied to make predictions on the test set.

For logistic regression, models with different levels of complexity were built. The model with the smallest Akaike information criterion (AIC) was then selected as the final model and evaluated by the test set(Gareth, Daniela, Trevor, & Robert, 2013).

Gareth, J., Daniela, W., Trevor, H., & Robert, T. (2013). *An introduction to statistical learning: with applications in R*. New York: Springer.
