# Supplementary figures and images for: Drug‐naïve first‐episode schizophrenia spectrum disorders: Pharmacological treatment practices in inpatient units in Hunan Province, China
Source: Early Interv Psychiatry. 2020 Sep 14;15(4):1010–8. doi: 10.1111/eip.13046 (PMC8359180; doi:10.1111/eip.13046)

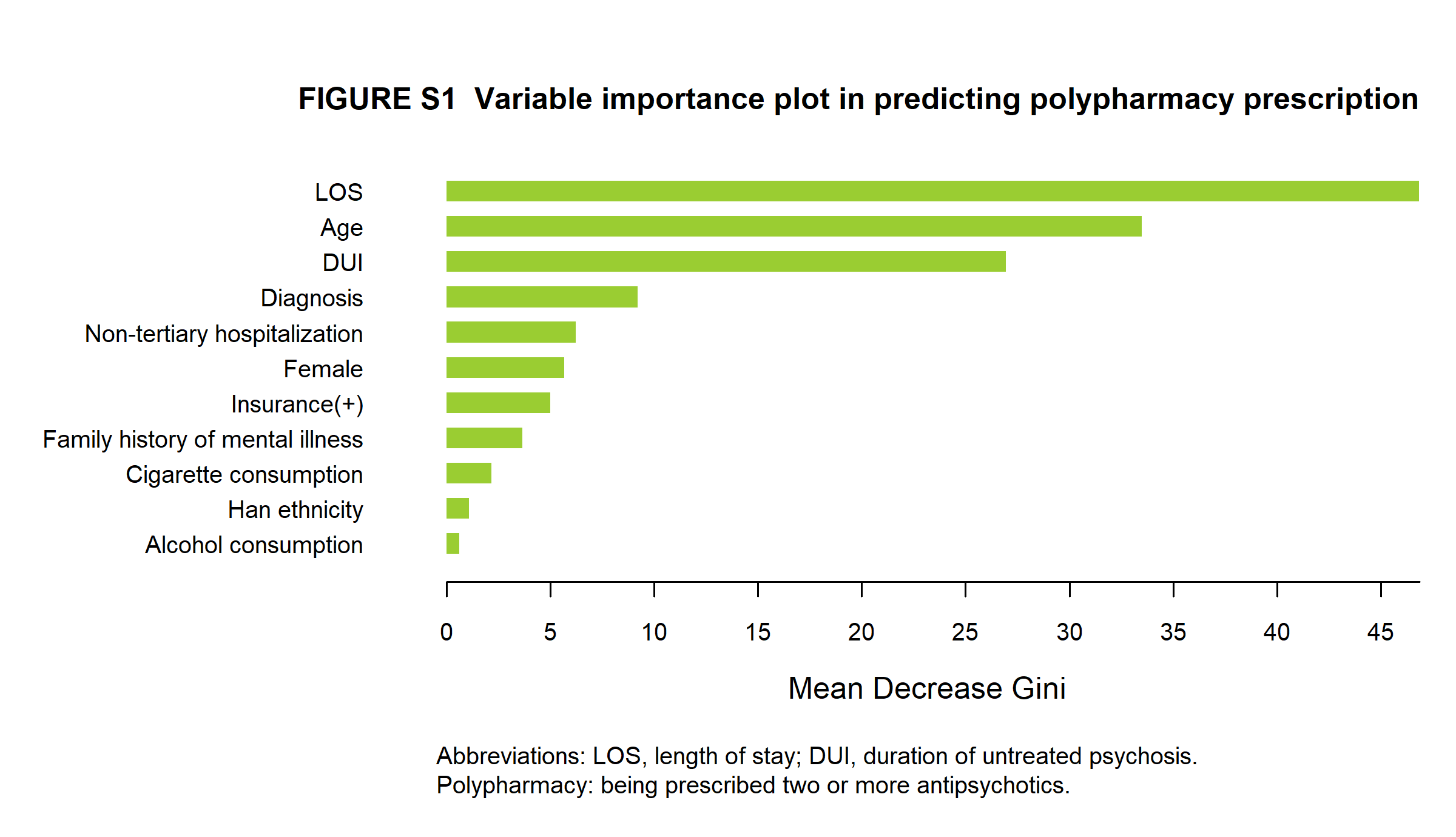

Supplement: Supplementary file 2 — Figure S1. Variable importance plot in predicting polypharmacy prescription. [file EIP-15-1010-s001.tiff]

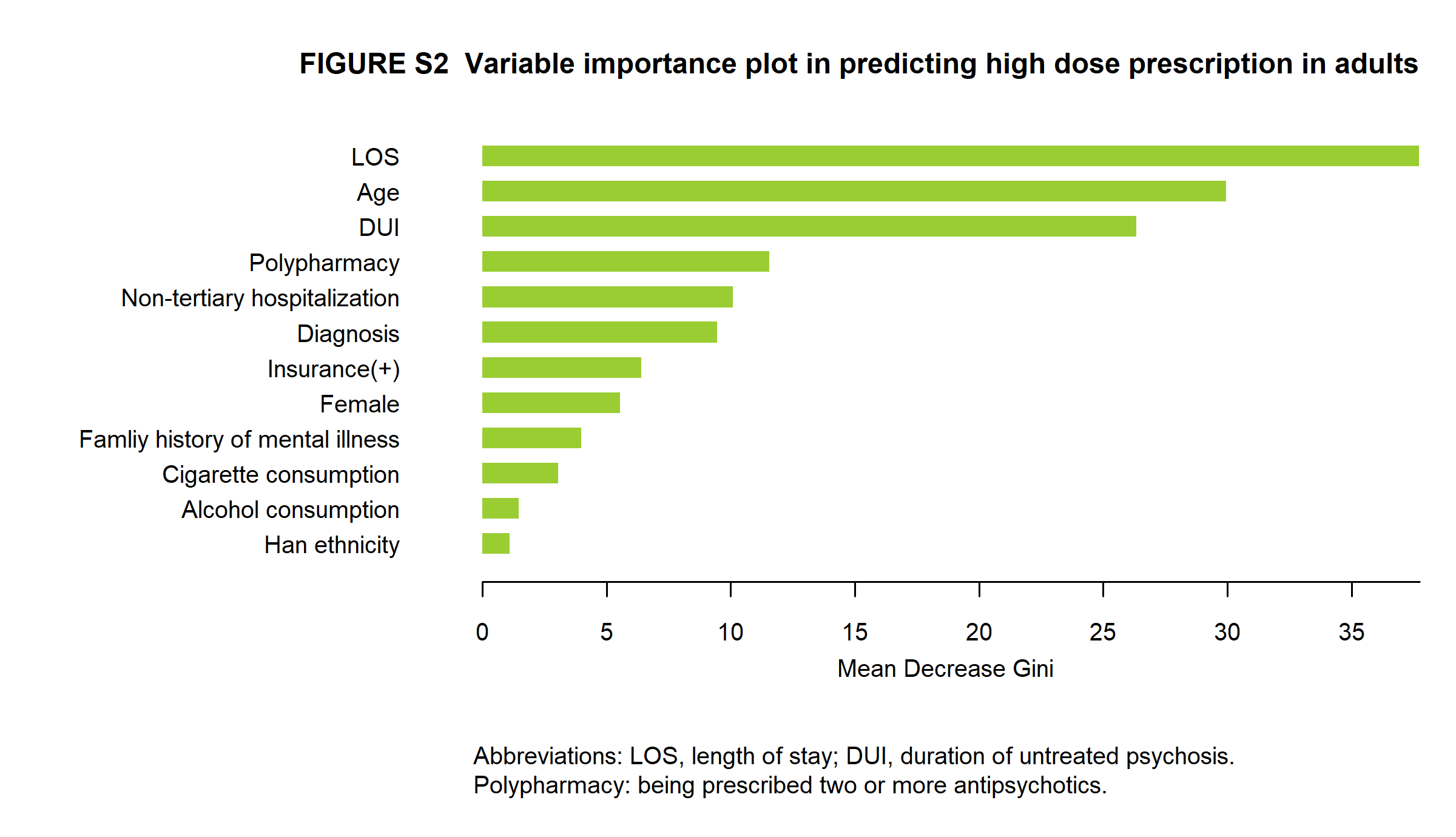

Supplement: Supplementary file 3 — Figure S2. Variable importance plot in predicting high close prescription in adults. [file EIP-15-1010-s007.tiff]

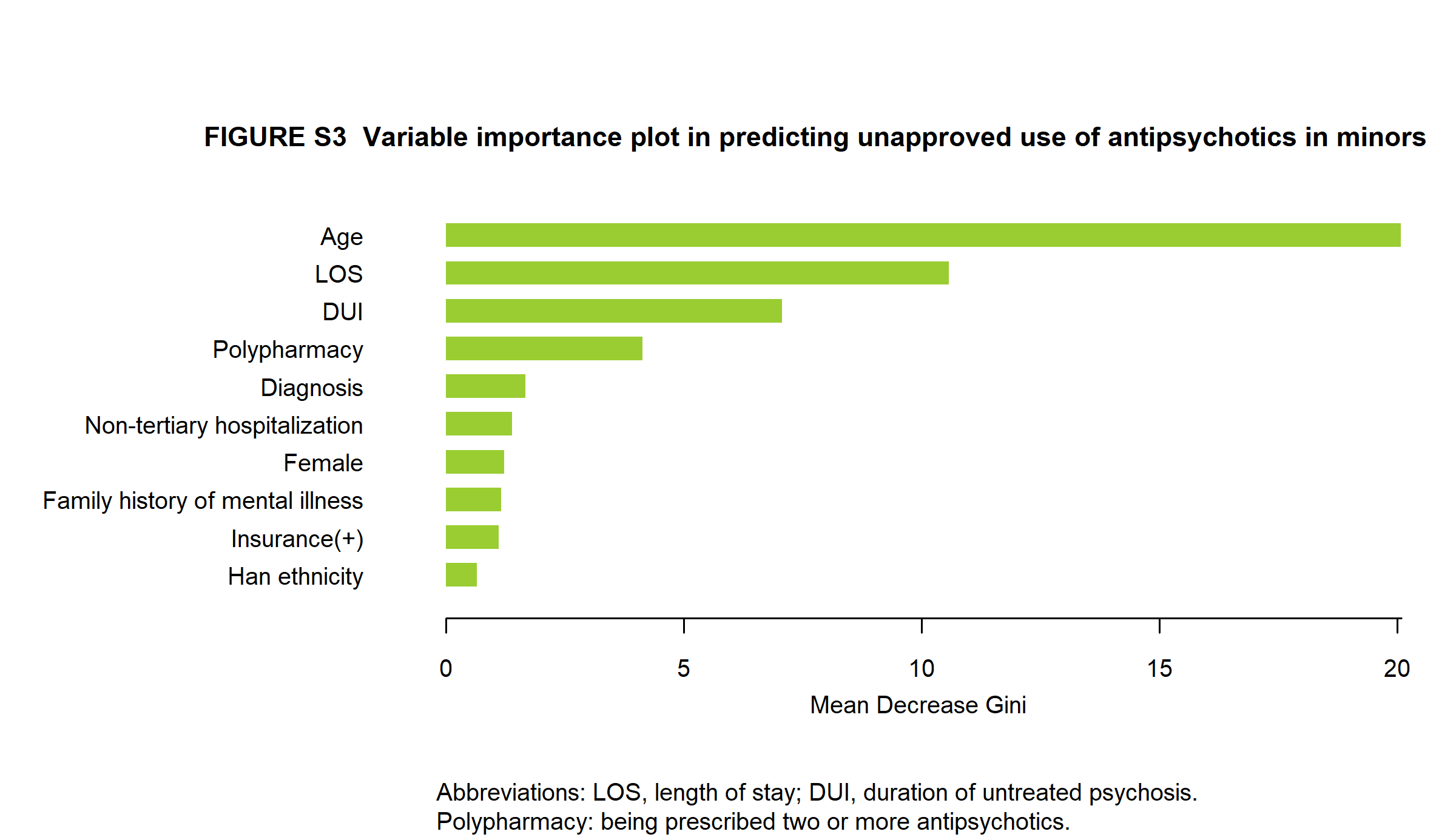

Supplement: Supplementary file 4 — Figure S3. Variable importance plot in predicting unapproved use of antipsychotics in minors. [file EIP-15-1010-s006.tiff]
