# Supplementary material for: Drug‐naïve first‐episode schizophrenia spectrum disorders: Pharmacological treatment practices in inpatient units in Hunan Province, China
Source: Early Interv Psychiatry. 2020 Sep 14;15(4):1010–8. doi: 10.1111/eip.13046 (PMC8359180; doi:10.1111/eip.13046)
Supplement: Supplementary file 5 — Table S1. Approved age range of antipsychotics for schizophrenia in China's guidelines. [file EIP-15-1010-s002.docx]

**TABLE S1** Approved age range of antipsychotics for schizophrenia in China’s guidelines

| **Generic Name** | **Approved Age Range for Schizophrenia by US FDA (Christian et al., 2012)** |
| --- | --- |
| FGAs |  |
| Chlorpromazine | 12 months to 12 years |
| Perphenazine | ≥ 12 years |
| Sulpiride | NA |
| SGAs |  |
| Amisulpride | NA |
| Aripiprazole | ≥ 13 years |
| Clozapine | ≥ 18 years |
| Iloperidone | ≥ 12 years |
| Olanzapine | ≥ 13 years |
| Paliperidone | ≥ 12 years |
| Quetiapine | ≥ 13 years |
| Risperidone | ≥ 13 years |
| Ziprasidone | ≥ 18 years |

Abbreviations: FGAs, first generation antipsychotics; SGAs, second generation antipsychotics; NA, not approved for marketing by US FDA(Bethesda, 2006-).

Bethesda. (2006-). *Drugs and Lactation Database (LactMed) [Internet]*. In. Retrieved from <https://www.ncbi.nlm.nih.gov/books/NBK501922/>

Christian, R., Saavedra, L., Gaynes, B. N., Sheitman, B., Wines, R. C. M., Jonas, D. E., . . . Carey, T. S. (2012). *Future Research Needs for First- and Second-Generation Antipsychotics for Children and Young Adults. Future Research Needs Paper No. 13*. Rockville MD: Agency for Healthcare Research and Quality.
