# Supplementary material for: Drug‐naïve first‐episode schizophrenia spectrum disorders: Pharmacological treatment practices in inpatient units in Hunan Province, China
Source: Early Interv Psychiatry. 2020 Sep 14;15(4):1010–8. doi: 10.1111/eip.13046 (PMC8359180; doi:10.1111/eip.13046)
Supplement: Supplementary file 6 — Table S2. Differences of factors in clozapine prescription. [file EIP-15-1010-s003.docx]

**TABLE S2**  Differences of factors in clozapine prescription

|  | **Clozapine (+) (n=45)** | | **Clozapine (-) (n=553)** | | **χ^2^/t** | **P-value** |
| --- | --- | --- | --- | --- | --- | --- |
|  | **N** | **%** | **N** | **%** |  |  |
| Adult (age≥18) | 35 | 77.8 | 408 | 73.8 | 0.170 | 0.681 |
| Male | 25 | 55.6 | 292 | 52.8 | 0.040 | 0.841 |
| Han ethnicity | 44 | 97.8 | 539 | 97.5 | NA^a^ | 1.000 |
| Public health insurance | 35 | 77.8 | 351 | 63.5 | 3.220 | 0.073 |
| Non-tertiary hospitalization | 30 | 66.7 | 237 | 42.9 | 8.606 | 0.003** |
| Polypharmacy^b^ | 33 | 73.3 | 88 | 15.9 | 81.488 | <0.001*** |
| LOS (week) (mean/SD) | 8.02/4.93 | | 4.35/4.18 | | -4.857 | <0.001*** |
| DUI (month) (mean/SD) | 4.75/4.45 | | 4.01/4.76 | | -1.069 | 0.290 |
| Family history (yes) | 4 | 8.9 | 71 | 12.8 | 0.256 | 0.613 |
| Cigarette consumption (yes) | 0 | 0.0 | 34 | 6.1 | NA^c^ | NA^c^ |
| Alcohol consumption (yes) | 0 | 0.0 | 11 | 2.0 | NA^c^ | NA^c^ |

Abbreviations: LOS, length of stay; DUI, duration of illness; NA, not applicable.

^a^ Fisher’s exact test was conducted due to not applicable for chi-square test.

^b^ Polypharmacy: being prescribed two or more antipsychotics.

^c^ Statistical tests not applicable.

**P<0.01. ***P<0.001.
