# Supplementary material for: Drug‐naïve first‐episode schizophrenia spectrum disorders: Pharmacological treatment practices in inpatient units in Hunan Province, China
Source: Early Interv Psychiatry. 2020 Sep 14;15(4):1010–8. doi: 10.1111/eip.13046 (PMC8359180; doi:10.1111/eip.13046)
Supplement: Supplementary file 7 — Table S3. Frequency of prescriptions deviating from guidelines (Chinese Society of Psychiatry affiliated with Chinese Medical Association, 2015). [file EIP-15-1010-s005.docx]

**TABLE S3** Frequency of prescriptions deviating from guidelines(Chinese Society of Psychiatry affiliated with Chinese Medical Association, 2015)

| **Type of deviation from guidelines** | **Adults (n=443)** | | **Minors (n=155)** | | | **Total (n=598)** | | |  |
| --- | --- | --- | --- | --- | --- | --- | --- | --- | --- |
|  | **N** | **(%)** | | **N** | **(%)** | | **N** | **(%)** | |
| Polypharmacy only | 45 | 10.1 | | 9 | 5.8 | | 54 | 9.0 | |
| High dose antipsychotics only | 93 | 21.0 | | NA | NA | | 93 | 15.6 | |
| Unapproved use of antipsychotics only | NA | NA | | 23 | 14.8 | | 23 | 3.8 | |
| Polypharmacy and high dose antipsychotics | 54 | 12.2 | | NA | NA | | 54 | 9.0 | |
| Polypharmacy and unapproved use of antipsychotics | NA | NA | | 13 | 8.4 | | 13 | 2.2 | |
| Total | 192 | 43.3 | | 45 | 29.0 | | 237 | 39.6 | |

Abbreviation: NA, not applicable.

Chinese Society of Psychiatry affiliated with Chinese Medical Association. (2015). *China’s Guidelines for Schizophrenia* (2nd ed.). Beijing: Chinese Medical Multimedia Press.
