# Supplementary material for: Drug‐naïve first‐episode schizophrenia spectrum disorders: Pharmacological treatment practices in inpatient units in Hunan Province, China
Source: Early Interv Psychiatry. 2020 Sep 14;15(4):1010–8. doi: 10.1111/eip.13046 (PMC8359180; doi:10.1111/eip.13046)
Supplement: Supplementary file 8 — Table S4 Dosage of single antipsychotic in monotherapya and polypharmacyb. [file EIP-15-1010-s004.docx]

**TABLE S4** Dosage of single antipsychotic in monotherapy^a^ and polypharmacy^b^

|  | **Monotherapy^a^** | | **Polypharmacy^b^** | | **P-value** |
| --- | --- | --- | --- | --- | --- |
|  | **Mean** | **SE** | **Mean** | **SE** |  |
| Amisulpride | 800.0 | 66.67 | 542.9 | 84.11 | 0.033* |
| Aripiprazole | 24.8 | 2.98 | 18.2 | 1.55 | 0.061 |
| Chlorpromazine | 500.0 | 0 | 125.0 | 21.13 | NA^c^ |
| Clozapine | 164.3 | 47.51 | 73.1 | 11.55 | 0.106 |
| Olanzapine | 15.9 | 0.38 | 11.0 | 0.81 | <0.001*** |
| Paliperidone | 8.0 | 1 | 9.0 | NA^d^ | NA^d^ |
| Quetiapine | 413.1 | 58.81 | 219.0 | 39.8 | 0.011* |
| Risperidone | 4.1 | 0.09 | 4.3 | 0.23 | 0.559 |
| Sulpride | 900.0 | 100 | 400.1 | 136.21 | NA^c^ |
| Ziprasidone | 160.0 | NA^d^ | 100.0 | 15.49 | NA^d^ |

^a^ Monotherapy: being prescribed one antipsychotic agent.

^b^ Polypharmacy: being prescribed two or more antipsychotics.

^c^ Sample size is not large enough to conduct t-test.

^d^ Only one observation in the sample.

*P< 0.05. ***P< 0.001
